# Scenarios for Optical Encryption Using Quantum Keys †

**DOI:** 10.3390/s24206631

**Published:** 2024-10-15

**Authors:** Luis Velasco, Morteza Ahmadian, Laura Ortiz, Juan P. Brito, Antonio Pastor, Jose M. Rivas, Sima Barzegar, Jaume Comellas, Vicente Martin, Marc Ruiz

**Affiliations:** 1 Advanced Broadband Communications Center (CCABA), Universitat Politècnica de Catalunya (UPC), 08034 Barcelona, Spain; 2 Electrical Engineering, Chalmers University of Technology, 41296 Gothenburg, Sweden; 3 Industrial Engineering School, Universidad Politécnica de Madrid, 28040 Madrid, Spain; 4 Telefonica Innovación Digital, 28050 Madrid, Spain

**Keywords:** optical encryption, Quantum Random Number Generator, Quantum Key Distribution, Post-Quantum Cryptography

## Abstract

Optical communications providing huge capacity and low latency remain vulnerable to a range of attacks. In consequence, encryption at the optical layer is needed to ensure secure data transmission. In our previous work, we proposed LightPath SECurity (LPSec), a secure cryptographic solution for optical transmission that leverages stream ciphers and Diffie–Hellman (DH) key exchange for high-speed optical encryption. Still, LPSec faces limitations related to key generation and key distribution. To address these limitations, in this paper, we rely on Quantum Random Number Generators (QRNG) and Quantum Key Distribution (QKD) networks. Specifically, we focus on three meaningful scenarios: In Scenario A, the two optical transponders (Tp) involved in the optical transmission are within the security perimeter of the QKD network. In Scenario B, only one Tp is within the QKD network, so keys are retrieved from a QRNG and distributed using LPSec. Finally, Scenario C extends Scenario B by employing Post-Quantum Cryptography (PQC) by implementing a Key Encapsulation Mechanism (KEM) to secure key exchanges. The scenarios are analyzed based on their security, efficiency, and applicability, demonstrating the potential of quantum-enhanced LPSec to provide secure, low-latency encryption for current optical communications. The experimental assessment, conducted on the Madrid Quantum Infrastructure, validates the feasibility of the proposed solutions.

## 1. Introduction

The high capacity of optical communications brought by higher-order modulation schemes, like Quadrature Amplitude Modulation (QAM), and their inherent low latency make them ideal for supporting 5G and beyond communications [1] from the transport core to the access network [2]. However, in standard optical communications, data are generally transmitted as plain text because implementing cryptographic methods to run at line speeds of hundreds of Gb/s is challenging, and their implementation might impact on the performance of the supported services, especially on the added transmission delay [1]. In addition, optical networks are themselves vulnerable to a variety of attacks such as eavesdropping, physical infrastructure attacks, interception, and jamming [3]. Therefore, quantum communications, providing superior security, continue their development and can finally provide the required capacity and performance [4] to replace classical optical communications, services must be individually secured at the packet layer using standard stream ciphers, like Advanced Encryption Standard [5] and ChaCha [6]. In parallel, quantum computing will soon be able to perform relevant cryptographic operations and break public cryptography. Therefore, advancing in the deployment of more secure communication infrastructures should be prioritized to secure the optical layer, so as to avoid the risks of building a secure platform on top of an unsecure one.

In our previous works [7,8], we proposed *LightPath SECurity* (LPsec), a secure cryptographic solution for point-to-point and point-to-multipoint optical transmission (e.g., 16-QAM) that: (*i*) extends optical transponders (Tp) with a fast bit stream Data Encryption Mechanism (DEM) using stream ciphers; (*ii*) implements key exchange based on the Diffie–Hellman (DH) [9] protocol orchestrated by the Software-Defined Networking (SDN) controller; and (*iii*) distributes symmetric keys through the encrypted optical channel. LPSec has shown its ability to provide data stream encryption at line speed, while introducing noticeable low processing delay.

Nonetheless, two main limitations can be found on LPSec: (*Lim-A*) *key generation*. This limitation refers to the use of a secure Pseudo-Random Number Generator (PRNG) to generate keys, which produces not truly random random-number sequences; and (*Lim-B*) *key distribution*. This limitation comes from the use of the previous key to encrypt the new key, which creates key dependency that can be exploited if one key is compromised. These limitations make LPSec to be not secure against the quantum threat.

To address these issues, in this paper, we propose using Quantum technologies, specifically: (*i*) Quantum Random Number Generators (QRNG) [10] to produce true-random numbers coming from inherently non-deterministic quantum processes, which solves *Lim-A*; and (*ii*) Quantum Key Distribution (QKD) networks [11,12], which provide an Information Theoretically Secure (ITS) protocol for distributing symmetric keys, thus facing *Lim-B*. In fact, although QRNG can be used on its own for classical processes, they are usually used inside the QKD devices. Hence, a QKD network can be understood as a network to generate and distribute truly random keys between any two points in the network. The combination of LPSec and quantum cryptography represents a step forward for the security of optical communications, as it allows upgrading technologies currently deployed in operators network with security resistant to attacks from quantum computing, thus extending their lifespan. The maturity of QKD devices and their commercial availability have allowed the deployment of experimental infrastructures (see, e.g., [13,14]). Furthermore, efforts devoted to the standardization of application interfaces for key retrieval (e.g., ETSI QKD 004 [15]), have eased QKD integration and enabled a wide-range of use cases.

However, although QKD can share infrastructure with classical optical communications, deploying a QKD network infrastructure is expensive and has distance limitations until quantum repeaters are commercially available. Therefore, in the application of QKD to LPSec, it will be very common that only one of the Tps of the optical connection is within the reachable perimeter of the QKD network, e.g., on optical connections between core/metro and access/aggregation sites. This situation entails reducing the security level in order to extend the optical network, since although standalone QRNGs can be used for key generation (thus solving *Lim-A*), key distribution mechanism still relies on LPSec, which keeps unsolved *Lim-B*.

A classical possibility to overcome the distance limitations and preserve resistance quantum attacks by Shor’s algorithm [16] is to use Post-Quantum Cryptography (PQC) algorithms, especially those providing a Key Encapsulation Mechanism (KEM). Since PQC algorithms are primarily based on the computational difficulty of solving certain mathematical problems, *Lim-B* can be unlocked. Recently, the National Institute of Standards and Technology (NIST) has published FIPS 203 [17], FIPS 204 [18], and FIPS 205 [19], which specify algorithms derived from CRYSTALS-Dilithium, CRYSTAL-KYBER and SPHINCS+ [20]. For our implementation, we have selected the Bit Flipping Key Encapsulation (BIKE) scheme [21]. BIKE is one of the earliest code-based cryptosystems and has proven resistant to quantum attacks by the Shor’s algorithm due to its NP-hardness in decoding random linear codes [22].

QKD and PQC are the solutions against cryptographic attacks via quantum computing. However, they both are complementary; QKD offers ITS, while PQC facilitates scalable authentication. In consequence, some previous works have proposed solutions involving QKD and PQC. Just to cite some very recent examples, the authors in [23] presented a quantum-secure architecture with PQC and QKD tailored explicitly for mobile networks and provided use cases that emphasize the need for advanced protection measures. An authenticated hybrid key exchange protocol that incorporates PQC and QKD and provides both forward and post-compromise security was introduced in [24]. The authors in [25] aimed to establish a secure system for key exchange by using PQC schemes in the classical channel of QKD to address authentication and encryption critical security challenges, so as to ensure reliable communication across quantum and classical channels, while the authors in [26] use PQC to provide encryption between KMS in two different domains. QKD and PQC hybridizations have been showcased experimentally, e.g., the authors in [27] proposed different methods of interconnecting three QKD testbeds in Europe using hybridizations of QKD and PQC algorithms.

In this paper, we explore the outlined scenarios for optical encryption using quantum keys where keys are: (*A*) retrieved from a QKD network, assuming that both Tps are inside the security perimeter of the QKD network; (*B*) retrieved from a QRNG in the case that only one of the Tps is inside that security perimeter, and distributed using LPSec to the other Tp; and (C) retrieved from a QRNG as in (B) but encapsulated using BIKE as KEM and exchanged to the other Tp using LPSec. In all cases, the retrieved keys leverage the true-random number properties of quantum physics. Such keys are then expanded to the desired line rate using a secure PRNG. For all the aforementioned scenarios, we assume that an optical connection between two Tps is setup using a SDN controller and both Tps connect to a local key manager (KM) in the case of QKD or just one of the Tps connect to a local key server in the case of relying on a QRNG. The workflows have been designed in the Horizon Europe ALLEGRO project [28] and their experimental assessment has been carried out on the Madrid Quantum Infrastructure (MadQCI) [14]. The rest of this paper is organized as follows. Section 2 first briefly introduces LPSec and presents in depth the three application scenarios that are the focus of this work. Section 3 describes the workflows for implementing the scenarios in practice and Section 4 presents the experiments carried out for the assessment of the solutions. Finally, Section 5 draws the main conclusions of this work.

## 2. Optical Encryption Using Quantum Keys

### 2.1. Brief Summary of LPSec

**Figure 1:**
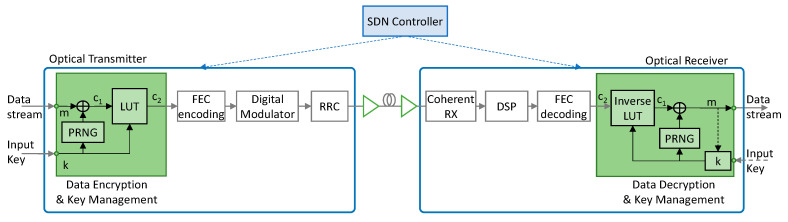
Optical coherent system implementing LPsec (extended from [7]).

LPsec [7] extends the standard coherent Tp with tailored solutions for both key exchange and encryption/decryption (see Figure 1). Cryptographic blocks operate at line speeds and therefore, optical encryption in LPSec is based on simple operations that can be easily implemented inside the optical Tp. For the key exchange, LPSec includes a mechanism based on the DH key exchange, where: (*i*) the initial public keys of the two end parties, i.e., the Transmitter (Tx) and the Receiver (Rx), are exchanged via the SDN controller; and (*ii*) keys are periodically updated through the optical channel to enhance the security level. As for the DEM, LPSec relies on two nested ciphers: (*i*) a stream cipher (*E_1_*(·)) that encrypts input data chunks *m* of predefined size with a key *kx*. Key *kx* is the result of expanding an input key *k* using a cryptographically secure PRNG. Encryption of *m* is performed is implementing as bitwise XOR operation, i.e., c_1_ = *E*_1_(*m*, *kx*) = *m* ⊕ *kx*, where *kx* and *m* have the same length; and (*ii*) a substitution cipher (*E*_2_(·)) for scrambling symbols. A Lookup Table (LUT) is used to create a ciphered gray map constellation through LUT permutations of incoming bits. The output ciphertext *c_2_* is produced by the combination of the inner stream cipher *E*_1_(·) and the outer substitution cipher *E*_2_(·), i.e., c_2_ = *E*_2_(*E*_1_(*m*, *kx*), *k*).

Each cipher has its drawbacks, but when combined they provide the required security level to encrypt data at 100 s of Gb/s exhibiting negligible transmission delay. For illustrative purposes, let us assume that input keys *k* are generated using the Xoshiro256+ PRNG, which is able to generate 256-bit keys in less than 1 ns using an Intel Core i7 [29]. After the input data are encrypted using stream cipher *E*_1_(·), the substitution cipher *E*_2_(·) adds more complexity to the brute-force attack, e.g., key spaces of 44 bits are produced using 16-QAM. That combination results in 300 bits key sizes, thus raising the effective computation required by the *birthday* paradox to over 2^150^, which can be considered safe enough for the foreseeable future [30]. This makes that LPsec can be considered secure against exhaustive search attacks.

However, (*i*) the sequence of stream keys generated by the PRNG from a given input key *k* cannot be infinite as this would reduce the security level; and (*ii*) the LUT permutation needs to be periodically regenerated to minimize vulnerabilities. As a result, the lifetime of keys is limited, e.g., to 1 s., which entails new keys being periodically made available at the two Tps. To facilitate communication between the two Tps, a special frame named Key exchange Frame (KxF) is used. A KxF is generated by the Tx and sent to the Rx periodically. The KxF includes a header of a fixed size that allows the Rx to detect its arrival.

### 2.2. Application Scenarios

In this work, we focus on the encryption of an optical signal, e.g., a 16-QAM signal, using the keys retrieved from a quantum key generation source. Retrieved keys are used in LPSec as input to the key expansion mechanism based on a secure PRNG to encrypt the input data stream at line rate. Then, three scenarios are analyzed depending on how keys are made available at the Rx side.

**Figure 2:**
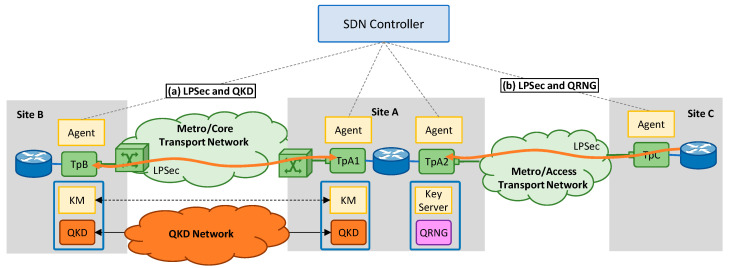
Scenarios under analysis: (**a**) both Tps are inside QKD security perimeter (e.g., in metro/core sites), and (**b**) one Tp is not within the security perimeter (e.g., in an access site) (**b**).

In the first scenario (*Scenario A*), both TpA1 and TpB are in sites covered by the QKD network (e.g., in metro/core sites), so keys *k* can be retrieved from the local KMs using a standard interface and used as input of the PRNG at both sides. In this scenario, (*i*) QKD is in charge of the generation and the distribution of truly random quantum keys; and (*ii*) the LPSec KxF header is used for synchronization purposes between the two Tps. Note that this is still needed to be sure that new keys are used for encryption and decryption at the right times. Figure 2a presents *Scenario A*, where the two Tps are co-located with QKD systems, each including its respective KM exposing an interface for key retrieval. An SDN controller is used for lightpath setup and LPSec configuration. Finally, two TP agents are responsible for configuring the local Tps and communicate with the SDN controller. *Scenario A* extends case 1 “undefined KSID in a single link scenario” defined in [15], to be used for LPSec connection setup.

In this scenario, the ITS security provided by the QKD network is reduced by the high expansion rate needed to expand retrieved key to the line rate, as QKD systems provide throughputs from hundreds of kb/s to few Mb/s, depending on the system.

To achieve the highest possible security level, both Tps need to be within the security perimeter of the QKD network, which is not always possible, e.g., when the optical connection involves the access network. In such cases, LPSec can be used to extend farther the reach of the security perimeter of the quantum network (*Scenario B*). Figure 2b depicts the scenario where only one of the TPs (TpA2) is in a metro/core site covered by the QKD network and a single QRNG is used as the entropy source. In this case, keys *k* can be retrieved by the local Tp from a local QRNG that provides a vendor-proprietary interface for key retrieval. Such random keys are used to encrypt the input data stream and exchanged to the remote Tp using the KxF header encrypted with the previous key. Typically, QRNGs have very high throughput (about 1 Gb/s), so moderate expansion is needed in this case to reach the desired line rate.

**Figure 3:**
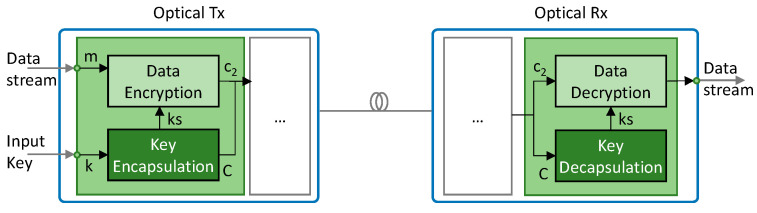
Extension of the optical system implementing LPsec DEM with BIKE KEM for Scenario C.

Note that in Scenario B, keys are used to encrypt the next keys that are distributed within the next KxF to be used during the next period. This creates a dependency that can be exploited to decrypt all the transmission if one of the keys is compromised. To avoid that, new keys, retrieved from a local QRNG, are used as part of key encapsulation (*Scenario C*). To that end, we modified the BIKE scheme to use the QRNG keys as seeds thus, replacing the PRNG in the encapsulation function. Random seeds together with the public key of TpC are inputs to generate the session key *ks* and the cyphertext *C*. Session keys *ks* are kept secret and used for data encryption, whereas cyphertexts *C* are exchanged with TpC that decapsulates them with its private key to retrieve *ks* and used for data decryption. Note that by doing so, we are ensuring higher randomness and increasing the overall security of the system. Figure 3 presents the modified optical coherent Tx and Rx that include both KEM and LPSec DEM.

## 3. Workflows

This section details the workflows to be implemented for each of the identified scenarios. The workflows start after the lightpath has been established over the optical network (step 0 in the workflows). LPSec requires an initial public key exchange to be carried out through the SDN controller as part of the provisioning process; the keys are used to generate the particular KxF header pattern that will be used on the optical channel between the two Tps. It is worth noting that details of the workflows have been intentionally omitted for the sake of simplicity; please refer to [7] for details related to LPSec, e.g., the use of public/private keys, KxF, and LUT.

### 3.1. Scenario A: LPSec DEM and QKD

**Figure 4:**
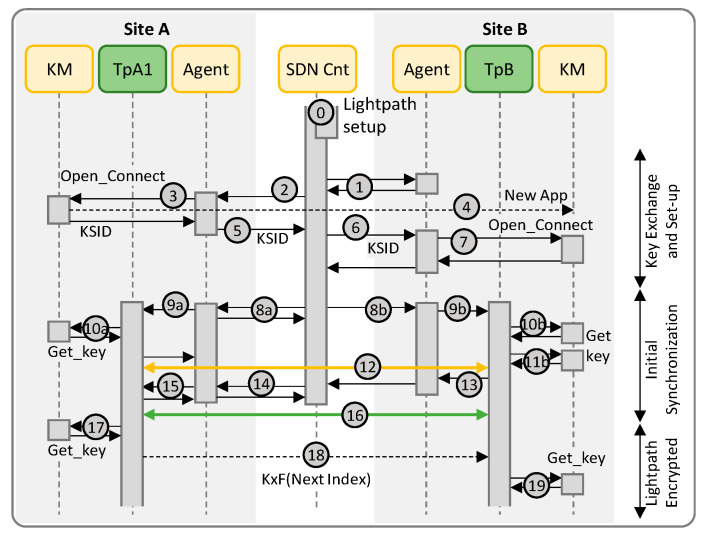
Workflow for Scenario A.

In Scenario A (Figure 4), the SDN collects the public key and the ID of TpB (labeled 1 in Figure 4) and sends them to the agent of TpA1 together with the details of the local KM (2). The agent in TpA1 connects to the local KM using the OPEN_CONNECT function and indicating the ID of TpA1 as source and that of TpB as destination. The KM replies with the *Key stream ID* (KSID) to be used for retrieving quantum keys (3). The KM in site A coordinates internally with the KM in site B to create the association KSID in both sites (4). The KSID needs to be used in site B, so TpA1 agent replies the SDN controller with it together with its public key, the ID of the local Tp and the KxF header pattern encrypted with the public key of TpB (5). The SDN controller, in turn, sends them to TpB agent (6). The agent in site B uses the KSID together with the IDs of the local Tp to connect to the local KM using the OPEN_CONNECT function (7) and reports to the SDN controller. At this time, both ends are ready to use the optical connection with encryption.

The next step is to synchronize the two Tps so they can start using the right keys to encrypt the data stream. Such initial synchronization is triggered by the SDN controller, which requests both ends to start the process (8a, 8b). In the case of the agent of TpA1, it acknowledges the request and asks the local Tp to get the first key (9a). TpA1 gets the key using the GET_KEY function with the obtained KSID (10a), uses it to encrypt a known bit stream and inserts the KxF header periodically including in the header the index of the retrieved key (12). Meanwhile, on site B, the agent sends the KSID and the ID of TpA1 to the local Tp (9b), which retrieves two keys (10b, 11b): the first key will be used during the synchronization period and the second once that period finishes and the real data stream is received. When TpB is able to successfully decrypt the known bit stream and finds the KxF header with the same index as the one got from the local KM, it notifies its agent that reports to the SDN controller (13). The SDN controller requests TpA1 agent to start (14), which in turns notifies the local Tp (15). At this time, both ends are ready to start with the real data encryption (16).

Once the Tps are synchronized, keys are updated periodically and synchronization is carried out using the KxF header. TpA1 gets the next key from the local KM (17), sends the index inside the KxF header (18) and starts encrypting the incoming data stream with the new key. Note that TpB already had the key for decrypting the data, so it uses that immediately, and gets the next key from the local KM (19). Steps 17–19 repeat at every time interval until the lightpath is torn down, when both agents use the KSID to terminate the association with the KMs.

### 3.2. Scenario B: LPSec DEM and QRNG

**Figure 5:**
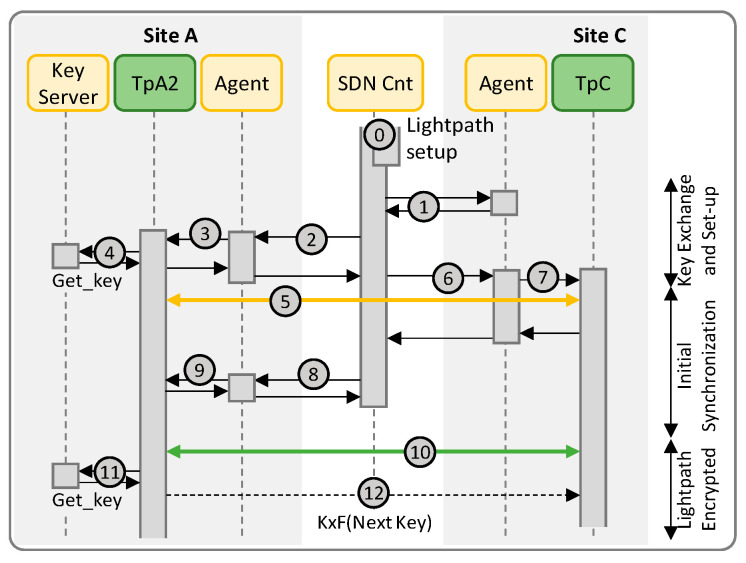
Workflow for Scenario B.

As in Scenario A, an initial public key exchange is carried out and used to generate the particular KxF header pattern that will be used on the optical channel between the two Tps for synchronization and key distribution. The SDN first collects the public key of TpC (labeled 1 in Figure 5) and sends it to TpA2 via its agent (2). The agent requests TpA2 to retrieve a key from the local key server that will be used for data encryption (3–4). As in Scenario A, TpA2 uses the retrieved key to encrypt a known bit stream and inserts the KxF header periodically (5). In addition, the key is sent to the SDN controller encrypted using the public key of TpC, together with the generated KxF header pattern, which are sent to TpC via its agent (6–7).

Once TpC is able to get synchronized with TpA2 through the optical channel, it replies the agent, which in turn notifies the SDN controller. Then, the SDN controller sends the confirmation to the agent in TpA2 (8), which notifies TpA2 (9) that the encryption of the incoming data stream can start (10).

Periodically, TpA2 retrieves a new key from the local key server (11), encrypts it using the previously retrieved key and sends it in the next KxF header (12). Steps 11–12 repeat at every time interval until the lightpath is torn down.

### 3.3. Scenario C: LPSec DEM, QRNG and PQC KEM

**Figure 6:**
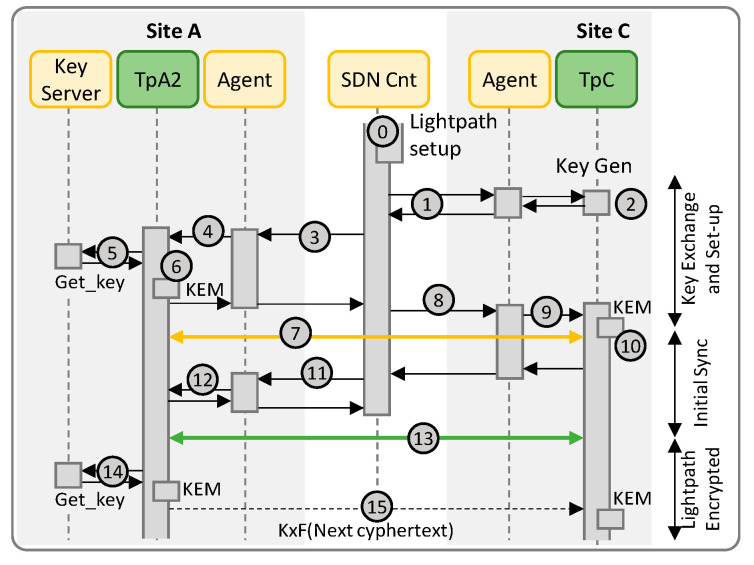
Workflow for Scenario C.

In this scenario, quantum keys are used in the PQC KEM to produce the session keys *ks* and the cyphertext *C* (Figure 6). Session keys *ks* are used as input to the DEM, while cyphertexts *C* are distributed between the Tps using the KxF header, which increases the security of the system. As in scenarios A and B, an initial public key exchange is carried out and used to generate the particular KxF header pattern. However, in this scenario, TpC generates its public-private keys specifically for the current lightpath. In addition, TpA2 needs the public key of TpC to produce the session key *ks* and the cyphertext *C*.

Therefore, when the SDN starts the workflow to collect the public key of TpC (labeled 1 in Figure 6), a new pair of public-private keys is generated by TpC (2) to be used to generate the KxF header pattern and the public key is collected by the agent that sends back in response to the SDN controller, which sends it to the agent of TpA2 (3). The agent requests TpA2 to retrieve a key from the local key server that will be used as a seed for data encryption (4–5). Specifically, TpA2 uses the retrieved seed to generate the session key and the cyphertext (6). The session key is used to encrypt a known bit stream and the cyphertext is added to the KxF header that is inserted periodically (7). In addition, the generated KxF header pattern and the cyphertext are sent to the SDN controller, which sent them to TpC via its agent (8–9). The cyphertext is then decapsulated using the private key of TpC to get the session key to decrypt the incoming optical signal (10).

The rest of the workflow is similar to that of Scenario B. Once TpC is able to get synchronized with TpA2 through the optical channel, it replies the agent, which in turn notifies the SDN controller. Then, the SDN controller sends the confirmation to the agent in TpA2 (11), which notifies TpA2 (12) that the encryption of the incoming data stream can start (13). Periodically, TpA2 retrieves a new key from the local key server (14) and uses it to generate a new session key and cyphertext. The new session key will be used to encrypt the data stream in the next period, while the cyphertext is sent in the next KxF header encrypted using the previous session key (15). Steps 14–15 repeat at every time interval until the lightpath is torn down.

## 4. Experimental Assessment

In this section, the setup used for the experimental assessment is first described and then, the workflows of the application scenarios described in the previous section are assessed.

### 4.1. Setup for the Experiments and Security Analysis

The setup in Figure 2 has been experimentally assessed using real QKD and QRNG systems. Specifically, a QKD link using experimental Huawei continuous-variable QKD devices [31] are used for Scenario A. The link connects two Telefonica’s facilities in Madrid, Spain; it spans 15 km and it has 10.2 dB losses to provide secret key rate of 8.4 Kb/s through an ETSI GS QKD 004 interface. In addition, a QuSIDE QRNG system [32] deployed in UPM premises is used for Scenarios B and C as quantum entropy source. The QRNG implements a proprietary phase-diffusion quantum random number generation technology and has embedded randomness metrology capabilities [33,34] to produce very high-quality random bits at 4 Gb/s, thus enabling a noticeable reduction in key expansion. The system exposes a proprietary REST API interface to deliver the random bits.

Using these systems to generate input keys *k* noticeably increases the security of LPSec. To illustrate this, Table 1 presents the total key size and the effective computation required by the brute-force attack for the plain LPSec using the Xoshiro256+ PRNG as key generator, and when LPSec uses keys from the Quantum systems described above. Note that the use of quantum systems makes the size space added by the substitution cipher *E*_2_(·) to be not significant and thus, it can be removed without affecting the security level of LPSec. This would noticeably simplify, and thus reduce the cost, of the optical transponders implementing LPSec.

The optical transmission, LPSec, including encryption and key exchange, as well as the modified BIKE scheme are implemented in a simulator running in UPC premises in Barcelona, Spain. The simulator has been developed in Python 3.10 and it is based on the architecture in [35], where several modules, including algorithms and interfaces communicate through a Redis DB in publish-subscribe mode. The setup was deployed inside Docker containers on an Ubuntu Server 22.04 LTS as operating system.

Finally, a video streaming service was implemented in Python 3.10 using the OpenCV library [36] to demonstrate the connectivity and data encryption. Due to the software implementation, the video streaming service was configured to run at 30 fps, which resulted in a smooth visualization in all the scenarios.

### 4.2. Assessment of the Application Scenarios

**Figure 7:**
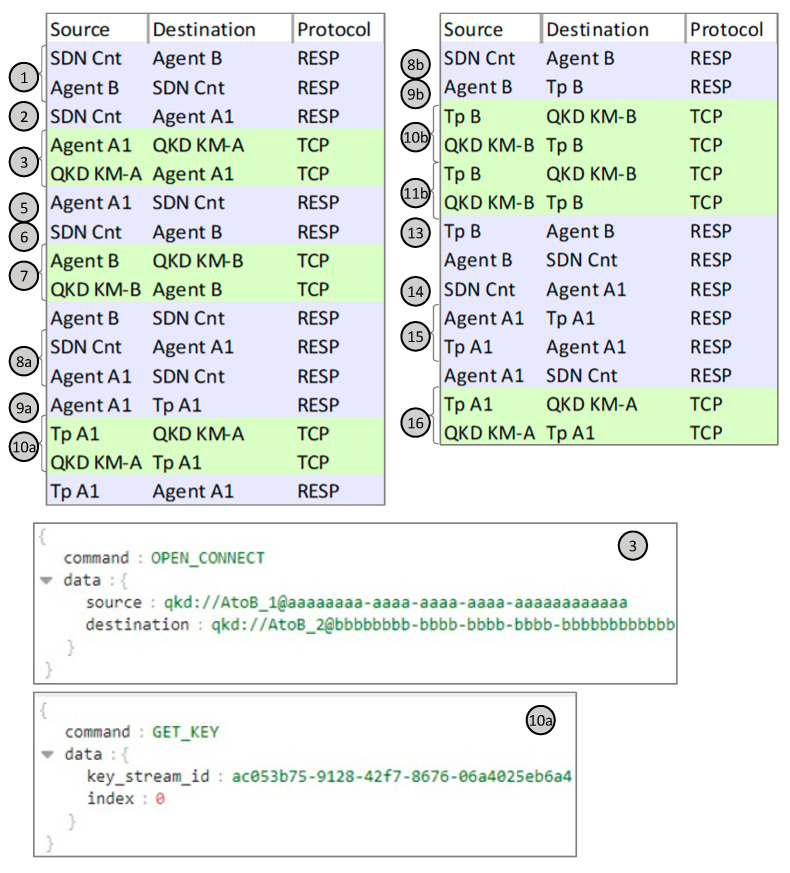
Capture of messages exchanged in the workflow for Scenario A and details of key messages.

Figure 7 reproduces the capture of messages exchanged between the different systems under Scenario A. Message exchange between modules implemented in the simulator is supported by the Redis serialization protocol (RESP). Actually, every message between two modules in the capture in Figure 7 is a summarization of two exchanged messages between the originating module and the Redis DB for publishing the message on a defined topic and between the Redis DB and the second module (which is subscribed to the topic) for message delivery. The numbering of the messages follows that of the workflow in Figure 4 to facilitate their identification. The details of messages exchanged between TpA1 and the KM in site A for OPEN_CONNECT (message 3) and GET_KEY (messages 10a/b, etc.) are also shown in Figure 7. The OPEN_CONNECT function requires as inputs the IDs of the application source (TpA1) and destination (TpB), as well as some Quality-of-Service parameters (in our tests, we configured the length of the keys to 8 bytes) and it returns a KSID. The subsequent GET_KEY calls get the obtained KSID together with the index of the key to retrieve.

**Figure 8:**
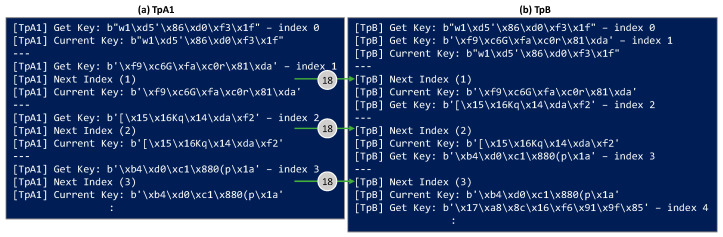
Outputs on terminals running TpA1 and TpB for Scenario A.

Figure 8 shows the log text in the terminals executing TpA1 and TpB. The keys and their indexes are printed when they are retrieved from the KMs. In this scenario, TpA1 obtains a KSID for the lightpath and shared it with TpB, so both Tps can get the keys from their local KM. TpA1 gets a key with the next index from the local KM and sends that index in the next KxF header. TpB gets two keys, one being the current and the other being the next one to be used as soon as a new KxF header is received specifying the next index in message 18. Then, a key with the next index is retrieved from the local KM. The capture shows the first three iterations.

**Figure 9:**
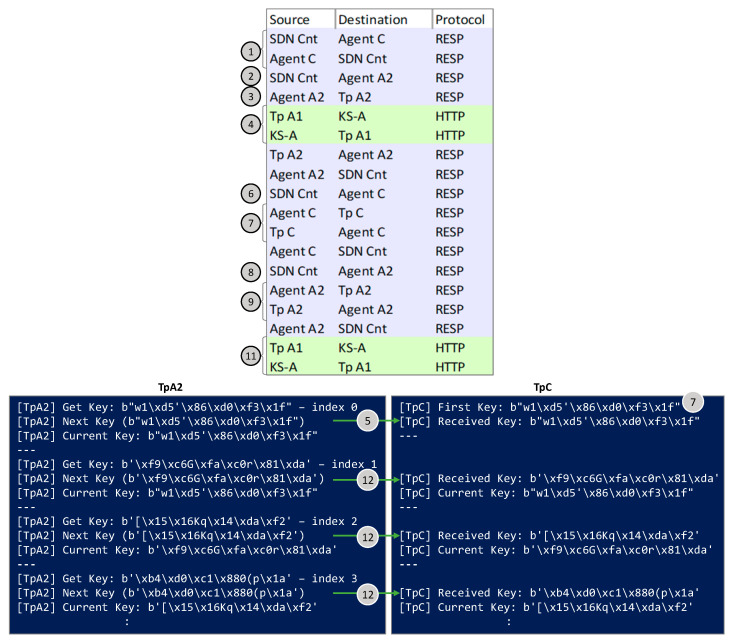
Capture of messages exchanged in the workflow and outputs on terminals running Tps for Scenario B.

Figure 9 presents the capture of the exchanged messages under Scenario B, as well as the log text on terminals running the Tps under Scenario B. In this scenario, the keys (8 bytes) are retrieved by TpA2 from the local QRNG and exchanged encrypted through the optical channel using the KxF header (messages 5 and 12). In addition, the first key is shared with TpC thought the control plane for the initial synchronization (message 7).

**Figure 10:**
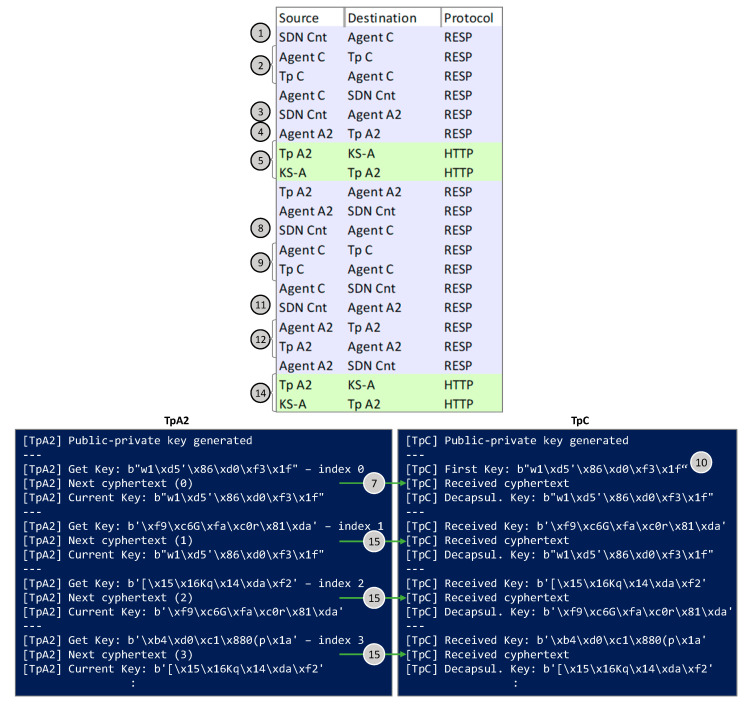
Capture of messages exchanged in the workflow and outputs on terminals running Tps for Scenario C.

Finally, Figure 10 shows the captures for Scenario C. A modified version of the BIKE scheme is used in: (*i*) both TpA2 and TpC to generate the pairs of public-private keys that will be used for the lightpath; (*ii*) in TpA2 to encapsulate the key retrieved from the QRNG and produce *ks* (8 bytes) and *C* (1549 bytes); and (*iii*) in TpC to decapsulate the received cyphertext and obtain *ks*. The first cyphertext is exchanged though the control plane (message 10) and used for the initial synchronization. In addition, TpA2 sends the initial cyphertext (message 7) and the subsequent ones (messages 15) within the KxF header though the optical channel.

## 5. Concluding Remarks

In this paper, we have explored the use of quantum solutions for improving the security of classical high-speed optical communications. In particular, we focused on improving LPSec, which has shown its ability to provide data stream encryption at line speed, while introducing noticeable low processing delay. However, as any classical cryptographic, LPSec is vulnerable to quantum attacks. Specifically, LPSec shows limitations in the way keys are generated and distributed. For that very reason, LPSec has been extended in this paper with truly random keys generated by quantum devices. The combination of LPSec and quantum security represents a clear benefit for network operators as it allows extending the lifespan of the optical technologies currently deployed in their networks, and therefore the proposed solutions have great potential for its deployment in current transport networks.

Three application scenarios have been experimentally assessed, differing in how the quantum keys are managed and distributed between the two Tps involved in the optical transmission. In Scenario A, the two Tps are within the security perimeter of the QKD network. In Scenario B, only one of the Tps is within the QKD network, so keys are retrieved from a QRNG and distributed using LPSec. Finally, Scenario C extends Scenario B by implementing a PQC KEM to secure key exchanges. A summary of the analysis is presented in Table 2. The table presents the main advantages and disadvantages of each application scenario, also highlighting their key functionalities and requirements in order to provide some insight of the applicability and practical application of the different proposals.

We have illustrated the scenarios with optical connections between metro/core sites that are assumed to be inside the Quantum security perimeter, and between a metro/core site and an access site, where the latter is assumed to be outside the security perimeter. Of course, the applicability of the solutions investigated in this paper is not restricted to those scenarios but it can be generalized to any other scenario depending on the availability of Quantum systems in one or the two end sites of the optical connection.

Overall, the investigated application scenarios show a practical and efficient way to combine classical and quantum optical technologies for enhancing the security of high-speed optical transmission. This represents a noticeable achievement of the ALLEGRO project [28], which aims at designing and validating a novel end-to-end sliceable, reliable, and secure architecture for next-generation optical networks, achieving high transmission/switching capacity.

## Figures and Tables

**Table 1:** Size of the keys and effective computation required by the brute-force attack.

	Size of *k* (bits)	Total Key Size (*E*_1_(·) + *E*_2_(·))	Effective Computation Required (*birthday* Paradox)
Plain LPSec	256	300	2^150^
Keys from QKD	8602	8646	2^4,322^
Keys from QRNG	4,194,304	4,194,348	2^2,097,174^

**Table 2:** Summary of the scenarios and comparative analysis.

Scenario	Description	Advantages	Disadvantages	Security Level
A	Both Tps are in the QKD network. Keys are retrieved directly from local KMs and expanded using a PRNG.	The highest security as keys are distributed using the QKD network. Direct key retrieval from KMs.	Large key expansion to match optical line speed (100 s Gb/s).	Highest
B	Only one Tp is within the QKD network. Keys are generated by a local QRNG and transmitted encrypted using the KxF header.	Extends the reach of the security perimeter of the quantum network. High throughput with QRNG. Moderate key expansion required.	Key dependency vulnerability if one key is compromised.	Medium/High
C	Only one Tp is within the QKD network. A modified BIKE scheme is used for KEM, ensuring higher randomness. Session keys are generated and cyphertexts are securely exchanged.	Extends the reach of the security perimeter of the quantum network. Increased overall security by removing key dependency vulnerability.	Complexity of PQC KEM.	Very High

## Data Availability

Data are contained within the article.
